# From Waste to Schiff Base: Upcycling of Aminolysed Poly(ethylene terephthalate) Product

**DOI:** 10.3390/polym14091861

**Published:** 2022-05-02

**Authors:** Ahmed A. Al Otaibi, Abdulmohsen Khalaf Dhahi Alsukaibi, Md. Ataur Rahman, Md. Mushtaque, Ashanul Haque

**Affiliations:** 1 Department of Chemistry, College of Science, University of Hail, Ha’il 81451, Saudi Arabia; a.alotaibi@uoh.edu.sa; 2 Experimental Research Building, Department of Chemistry, New York University Abu Dhabi, Abu Dhabi P.O. Box 129188, United Arab Emirates; 3 Department of Chemistry, School of Physical and Molecular Sciences, Al-Falah University, Dhauj, Faridabad 121004, India; mush.chem@gmail.com

**Keywords:** aminolysis, poly(ethylene terephthalate), Schiff base, valorization

## Abstract

Recycling plastic waste into valuable materials is one of the contemporary challenges. Every year around 50 million tons of polyethylene terephthalate (PET) bottles are used worldwide. The fact that only a part of this amount is being recycled is putting a burden on the environment. Therefore, a technology that can convert PET-based waste materials into useful ones is highly needed. In the present work, attempts have been made to convert PET-based waste materials into a precursor for others. We report an aminolysed product (3) obtained by aminolysis reaction of PET (1) with 1,2 diaminopropane (DAP, 2) under solvent and catalytic free conditions. The highest amount of monomeric product was obtained upon heating the mixture of diamine and PET at 130 °C. The resulting aminolysed product was then converted to a Schiff-base (5) in 25% yield. The chemical structure of the synthesized compounds was confirmed using multi-spectroscopic techniques. The results of this study will be a valuable addition to the growing body of work on plastic recycling.

## 1. Introduction

The Earth’s population is growing exponentially, and so are the synthetic pollutants. For instance, plastics (synthetic polymeric materials) are well-known ocean/land pollutants with low biodegradability and toxicity [1,2]. As per a report, 5–17% of the total solid waste generated in Saudi Arabia is mainly composed of plastic materials [3]. Among six major categories of plastics (as per the US Environmental Protection Agency), polyethylene terephthalate (PET) is one of the most common household names. It is used for the manufacture of water/soft drink bottles, food containers, fibers, etc. [4,5]. Folks worldwide use ~50 million tons of PET bottles annually [5]. Unfortunately, this extensive usage of PET also contributes to environmental pollution [6]. To eliminate this issue, both physical/mechanical and chemical recycling of PET has been suggested [5,7,8,9]. Though the mechanical method has its own advantages, there is an upsurge in developing a chemical recycling method. In literature, various chemical methods for PET depolymerization have been reported [5]. This includes, but is not limited to, aminolysis, hydrolysis, methanolysis, glycolysis, etc. [10,11,12,13,14,15]. Among these, aminolysis is particularly interesting due to the high reactivity (nucleophilicity) of amine leading to a faster reaction with PET [16]. Several examples of aminolysis reactions using a diamine or amino-alcohol have been reported with or without using a catalyst [17,18,19,20,21].

Researchers also demonstrated that the depolymerized product(s) could be used as feedstock/precursor to prepare functional materials [8,22,23]. Recently, Chan and Zinchenko [24] reported the conversion of PET to cross-linked hydrogels as an adsorbent for the cationic dye. Using various amino alcohols and an organic catalyst, Demarteau et al. [16] reported depolymerization of PET in a high yield and rapid manner. The resulting diols were then converted to low to moderate molecular weight poly(ester-amide)s. Rwei and co-workers [25] adopted alcoholysis and aminolysis reactions to prepare bis(6-aminohexyl) terephthalamide (BAHT), which was then used to prepare polyamide 66 (PA66) copolymers. The resulting copolymers were found to exhibit features better than or comparable to PA66. Motivated by these works and to further explore the application of aminolysed products, we report herein aminolysis of PET (1) with 1,2 diaminopropane (2). The depolymerization reaction was carried out without any solvent or catalyst. The obtained product (3) was then converted to a Schiff base (5) in reasonable yield. The chemical structure of the final products was characterized using multi-spectroscopic techniques. Overall, by the transformation of PET to oligomeric amides, we can use waste PET bottles as a source of materials and hence contribute to the chemical methods of recycling waste materials.

## 2. Materials and Methods

All reactions were conducted under open air conditions. All chemicals were procured from Sigma-Aldrich (St. Louis, MI, USA) and used without further purification. A commercially available water bottle was used. ^1^H and ^13^C NMR spectra were recorded on a Bruker Spectrospin DPX 500 MHz and 125 MHZ spectrometer, respectively, using trimethylsilane (TMS) as an internal standard. Splitting patterns are designated as follows: s, singlet; d, doublet; m, multiplet. Chemical shift values are given in ppm. Liquid chromatography/mass spectrometry (LC/MS) was performed on an Agilent LC/MS instrument (1260 Infinity II) equipped with a reverse-phase C_18_ column (2.7 μm particle size, 3.0 × 100 mm), electrospray (ESI) mass spectrometry detector, and photodiode array detector. Flash-column chromatography was performed using silica gel (60 Å, 40–63 μ).

### 2.1. Synthesis and Characterization

#### 2.1.1. Depolymerization of PET

The depolymerization of PET was carried out under the following reaction conditions (Table 1): In a round bottom flask, a mixture of 3.00 g of PET flakes and 30 mL (10 volume) of 1,2-diaminoprapane (DAP) was taken and heated at 100 °C (for four hours and then at room temperature for 20 h, method A) or 110 °C (for 24 h, method B) or 130 °C (for 24 h method C). In addition, 50 mL of methanol was then added to the resulting mixture for 3 h. Afterwards, the reaction was stopped, cooled to room temperature, and filtered. In the case of methods A and B, we noticed the presence of some insoluble materials which were carefully rinsed with methanol and acetone. The solvent and volatile materials in the combined filtrate were removed using a rotary evaporator, yielding a sticky liquid that was dried under a high vacuum for 24 h at 60 °C. However, in method C, a clear yellowish-green solution resulted that was cooled, filtered, and concentrated to yield a viscous liquid that was dried under a high vacuum. Leaving this liquid for a longer period resulted in a solid product. The final product was characterized using FTIR, NMR, and HRMS techniques. The following data for the product were obtained by method C: ^1^H-NMR (500 MHz, D_2_O) δ(ppm): ^1^H-NMR (500 MHz, D_2_O) δ 7.74 (d, J = 15.6 Hz, 4H), 3.62 (s, 4H), 3.30–3.18 (m, 4H), 3.11–2.98 (m, 2H), 1.05 (d, 6H). ^13^C-NMR (126 MHz, D_2_O) δ(ppm): 169.72 (Ar–CO–NH–), 136.54, 127.36 (aromatic carbon), 62.46 (–CH_2_–NH–), 45.67 (NH_2_–CH–CH_3_), 19.52(–CH_3_–). ESI-MS calculated C_14_H_23_N_4_O_2_, = *m*/*z* 279.17; observed = *m*/*z* 279.18 [M + 1].

#### 2.1.2. Synthesis of N^1^,N^4^-Bis(2-(((E)-2-hydroxybenzylidene)amino)propyl)terephthalamide (5)

At room temperature, salicylaldehyde (277 mg, 2.266 mmol in 5 mL ethanol) was added dropwise to an ethanolic solution of N,N-bis-(2-aminopropyl)-terephthalamide (3) (300 mg, 1.079 mmol in 10 mL ethanol) and, subsequent to this, the reaction mixture was heated at 70 °C overnight. Upon the completion of reaction (as indicated TLC), the solvent was evaporated under reduced pressure to yield the crude product. The obtained reaction mixture was purified using flash chromatography to afford Schiff base (5) (132 mg, 25% yield) as a yellow solid. In addition, another condensation product (6) (326 mg, 62% yield) was also obtained as a yellow gummy liquid: ^1^H-NMR (500 MHz, CDCl_3_) δ 13.25 (s, 2H), 8.38–8.27 (m, 2H), 7.62 (d, J = 7.5 Hz, 3H), 7.34–7.24 (m, 3H), 7.24–7.15 (m, 3H), 6.92 (tt, J = 8.1, 5.2 Hz, 2H), 6.85 (ddt, J = 9.1, 7.7, 6.0 Hz, 2H), 6.75–6.64 (m, 1H), 3.89–3.77 (m, 2H), 3.77–3.63 (m, 3H), 3.38–3.27 (m, 1H), 1.35 (d, 6H). ^13^C NMR (126 MHz, CDCl_3_) δ 166.90, 166.33, 164.89, 164.43, 160.89, 136.91, 132.54, 132.33, 131.46, 127.12, 118.83, 118.60, 118.48, 116.92, 116.87, 65.77, 64.97, 63.93, 46.29, 20.39, 20.01. ESI-MS calculated C_28_H_30_N_4_O_4_ = *m*/*z* 486.22; observed = *m*/*z* 487.22 [M + 1].

## 3. Results and Discussion

### 3.1. Aminyloysis of PET

**Figure 1:**
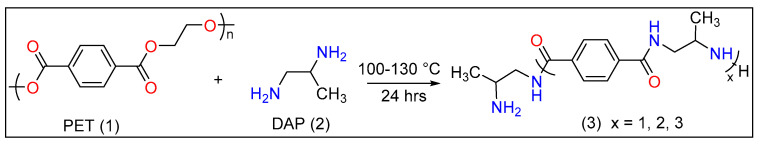
Chemical conversion of PET to corresponding amide.

Aminolysis of PET is well known in literature [26,27]. In the present work, we adopted a reported protocol to perform aminolysis of the PET flakes [11]. Briefly, PET was cut into small pieces, washed, and dried. In addition, 3.0 g of this finely cut PET (1) and 1,3-dimanopropane (DAP, 2) (30 mL) was heated at 100–130 °C (Figure 1 and Supplementary Material).

**Figure 2:**
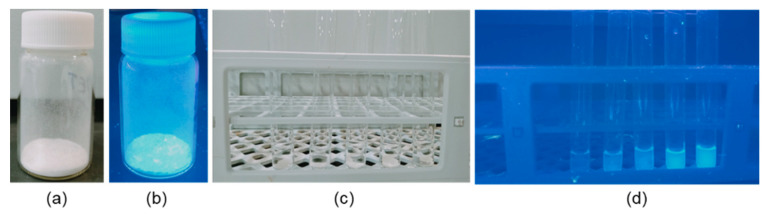
Images of solid aminolysed product (3) under normal (**a**) and UV light (**b**). Aqueous solution of (3) under normal (**c**) and UV-light (**d**). Solutions were prepared by dissolving a different amount (1, 2, 5, 10, 25 mg/mL) of (3) in distilled water.

To check the influence of temperature and reaction time, we conducted the same reaction under slightly different conditions (Table 1). When the reaction was carried out at 100 °C for four hours followed by stirring at room temperature (for 20 h), the product obtained was the mixture of monomer, dimer, trimer and other oligomers, as suggested by NMR and MS analysis (*vide infra*). In the second set of reactions, increasing the temperature (110 °C) and duration (24 h heating) led to the formation of short chain oligomers with monomer as one of the major products. Based on these two observations, we further raised the temperature to 130 °C and found that the major product in this case is mainly monomer (as indicated by water solubility and NMR analysis). After completion of the reaction, 10–15 mL of methanol was added to the reaction mixture and refluxed for an additional 3 h after. Upon cooling, a yellowish green, fluorescent liquid was obtained (in the case of method C). It is worth mentioning that the obtained product was highly soluble in water (even the solidified product). High solubility of the compound could be attributed to the presence of polar functionalities (amides and amines) and small size of the final product. This observation contrasts with the reaction with ethylene diamine (EDA) where a solid (large oligomers) and a liquid (small oligomers) is obtained [11]. In addition, compared to the related aminolysis product (with EDA), we found that the presence of an additional methyl substituent led to an emissive product with excellent water solubility (Figure 2). In a recent work, Chan and Zinchenko [24] also reported water soluble aminolysed products of PET.

### 3.2. Schiff-Base (5) Formation

**Figure 3:**
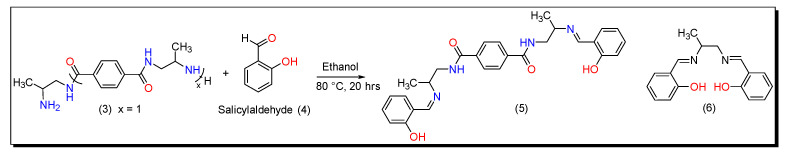
Chemical conversion of aminolysed product (**3**) to a Schiff base (**5**).

Schiff bases serve as an excellent precursor in the development of biologically active ligands, complexes, catalysts, etc. [28]. To explore the utility of the aminolysed product (3), we performed chemical modification at the –NH_2_ group by reacting with salicylaldehyde (4) (Figure 3). The resulting Schiff base (5) along with a side product (6) was obtained in reasonably good yield and purity. Analytical data suggest that the product mainly contained a Schiff base arising from the monomer unit (for analytical details, see Supplementary Material). The fact that compound (5) possesses coordinating sites for the metal chelation could be used to prepare complexes for different applications [29,30].

### 3.3. Infra-Red Studies (IR) Studies

**Figure 4:**
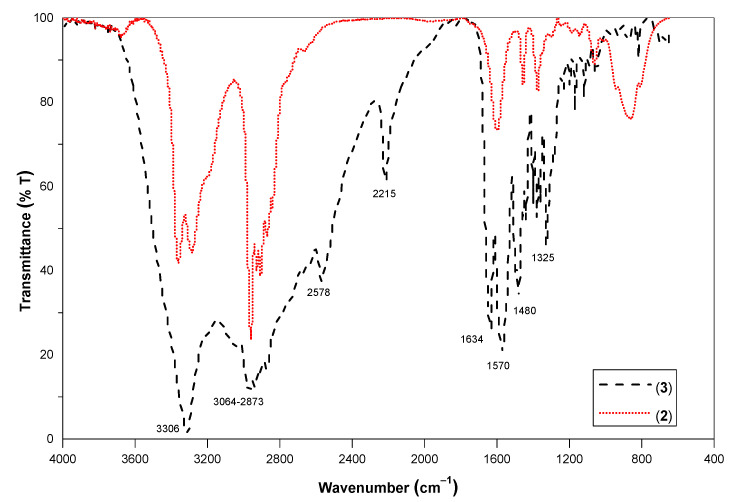
FTIR (ATR) spectra of (2) (red short dot line) and aminolysed product (3, black dash line) obtained by method C.

FTIR (ATR) spectra of (2) (red line) and aminolysed product (3, black line) are depicted in Figure 4. In the ATR spectrum of (3), peaks at 1634, 1570, and 3306 cm^−1^ can be attributed to –C=O_(str.)_, –NH_(def.)_, and –NH_(str.)_ bands, respectively. The later peak (at 3306 cm^−1^) as a single sharp peak confirms the presence of a secondary amide group. Stretching between 3064–2873 cm^−1^ is due to the aliphatic and aromatic C–H stretching. Lack of peaks at >1700 cm^−1^ supported the complete conversion of ester moieties to amide [31].

### 3.4. Nuclear Magnetic Resonance (NMR) Analysis

#### 3.4.1. NMR Analysis of Aminolysed Products

**Figure 5:**
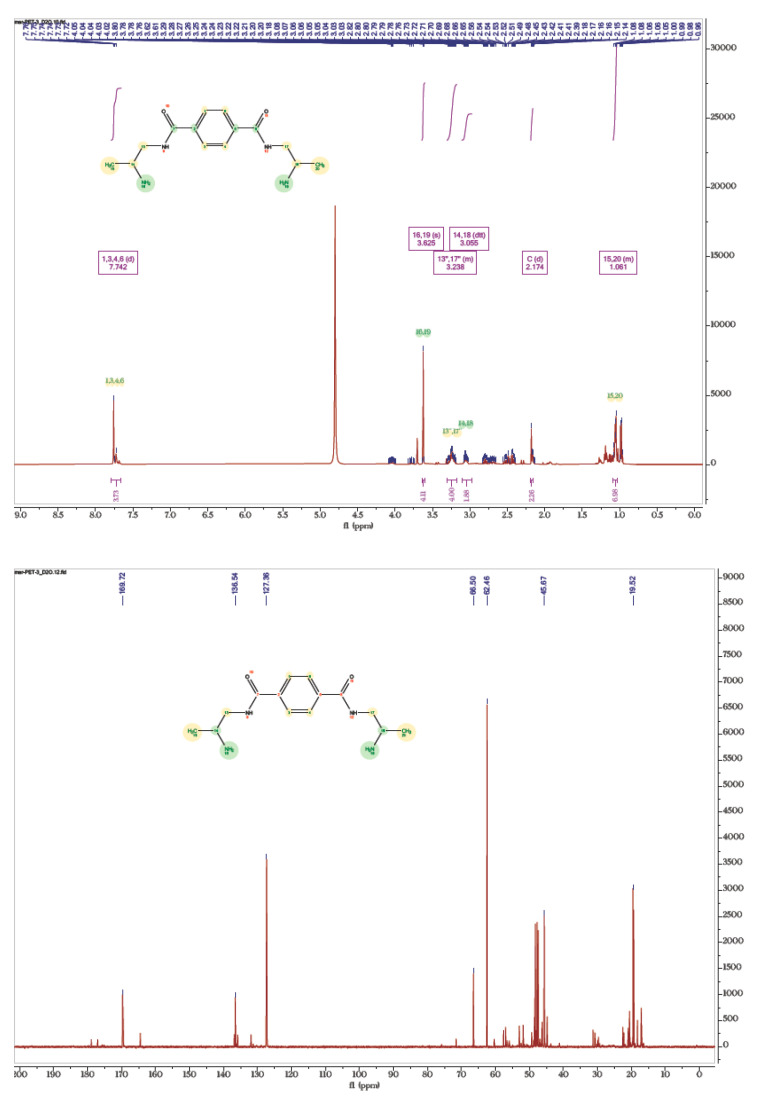
^1^H and ^13^C NMR spectra of aminolysed product (3) obtained by method C.

^1^H and ^13^C NMR spectra of the products obtained by method C are depicted in Figure 5, while those obtained by methods A and B are given in Figures S1–S4 (Supplementary Material). ^1^H-NMR spectrum (Figure 5, top) of compound (3) showed a doublet for aromatic (δ 7.74 ppm), a singlet for primary amine (δ 3.62 ppm), a multiplet for methylene (δ 3.23 ppm), and methine (δ 3.05 ppm) protons in the expected region. However, all protons in the proposed structure were present, and we could not see amide (–NH) protons due to the solvent exchange with D_2_O. In addition, the absence of downfield aromatic peaks and upfield methylene protons owing to polyester moiety further confirmed the depolymerization reaction [24]. Additional peaks~δ 4.0–4.1 ppm could be attributed to diethylene glycol (HO–CH_2_–CH_2_–OH) formed during the reaction. ^1^H-^1^H correlation spectroscopy (COSY) also supported the structure of the monomeric product. For instance, cross peaks showing correlation between methine and methyl protons, methine and methylene protons, etc. can be easily observed. In ^13^C NMR, amide of the carbonyl appeared at δ 162 ppm with aromatic carbons in the range of 136.5–127.4 ppm. Similarly, methine and methylene carbon centers are observed in the range of 62–19.5 ppm [24].

#### 3.4.2. NMR Analysis of the Schiff Bases

**Figure 6:**
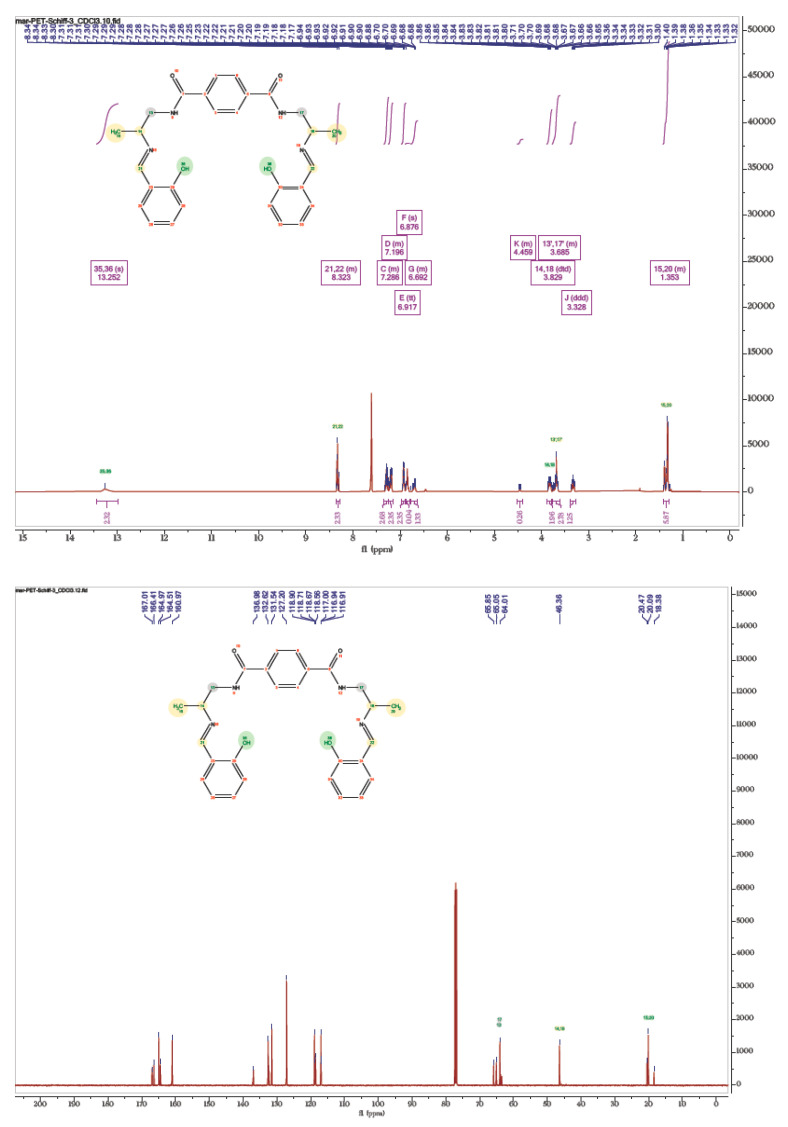
^1^H and ^13^C NMR spectra of the Schiff base (5).

The NMR spectra of the Schiff base (5) is shown in Figure 6. Due to the formation of imine linkage, –NH_2_ protons disappeared and a broad peak at δ 13.25 ppm (2 × –OH) appeared. Downfielded value of the hydroxyl proton could be attributed to the intramolecular H-bonding between O–H and N-atoms. A peak at δ 8.38–8.27 ppm is due to the azomethine proton (–N=C–H) unit. In addition, other peaks (*vide infra*) can also be seen in the spectrum. In the ^13^C spectrum, a set of peaks between δ 166.90–160.89 ppm can be ascribed to the carbonyl and azomethine carbons. All of these observations are well supported by two-dimensional spectroscopy. In addition to the above-mentioned Schiff base, we also obtained compound (6) as the side product. Structural analysis of this product revealed compound (6) as the Schiff base formed by the reaction of (2) and aldehyde (4). A complete characterization data for this compound is given in Figure S4 (Supplementary Material).

### 3.5. High Resolution Electrospray Ionization Mass (ESI-HRMS) Spectrometry

**Figure 7:**
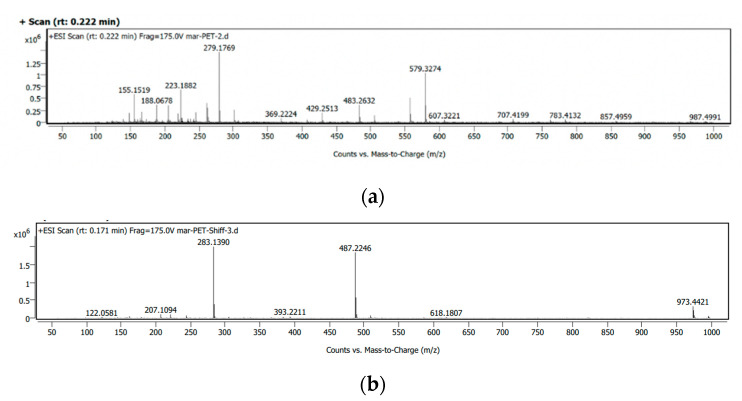
ESI-HRMS of aminolysed product obtained by method C (**a**) and the Schiff base (**b**).

ESI-HRMS data of the products obtained by method C are shown in Figure 7a, while the main peaks are tabulated in Table 2. ESI-HRMS data of the products obtained by method A and B are given in Figures S2 and S3 (Supplementary Material). Peaks at *m*/*z* 279.1, 483.2, and 707.4 corresponding to monomeric (C_14_H_22_N_4_O_2_), dimeric (C_25_H_34_N_6_O_4_), and trimeric (C_36_H_46_N_8_O_6_) units are respectively observed in the spectrum. As compared to the products obtained by method A & B, method C yielded monomer as the major component. Figure 7b shows the spectrum of the Schiff bases (5) at *m*/*z* 487.2 and (6) at *m*/*z* 283.1 formed by the condensation of monomer and DAP, respectively.

## 4. Conclusions

In this study, a new aminolysed product (3) was obtained by the reaction of poly(ethylene terephthalate) (1) and 1,2 diaminopropane (2) under solvent and catalytic free conditions. The resulting water-soluble amide was then subjected to a condensation reaction with salicylaldehyde (4) to yield a Schiff base (5) in reasonable yield and purity. Since the reaction was conducted without any solvent and at a moderate temperature, it may help to significantly broaden the scope of possible applications of PET waste. In addition, it also opens the door for the development of new compounds starting from waste materials.

## Figures and Tables

**Table 1:** Reaction conditions and major products obtained during aminolysis.

Method	Reaction Conditions	Major Product(s) ^1^
A	100 °C (4 h) → RT (20 h)	Monomer, *Dimer*, *Oligomers*
B	110 °C (24 h)	*Monomer*, *Dimer*
C	130 °C (24 h)	*Monomer*, Dimer

^1^ major product identified are in *italics*.

**Table 2:** Molecular formula and mass of the aminolysed products.

Unit	Mol. Formula	Mol. Wt. (Calculated)	Mol. Wt. (Observed)
Monomer	C_14_H_22_N_4_O_2_	278.3	279.1
Dimer	C_25_H_34_N_6_O_4_	482.5	483.2
Trimer	C_36_H_46_N_8_O_6_	686.8	707.4

## Data Availability

Not applicable.

## Supplementary Materials

The following supporting information can be downloaded at: https://www.mdpi.com/article/10.3390/polym14091861/s1, Figure S1: ^1^H-NMR (a), ^1^H-^1^H COSY (b) and ESI-MS (c) spectra of (3) obtained by method A; Figure S2: ^1^H-NMR (a), ^1^H-^1^H COSY (b) and ESI-MS (c) spectra of (3) obtained by method B; Figure S3: ^1^H-NMR (a), ^1^H-^1^H COSY (b), (c) ^13^C and ESI-MS (c) spectra of (3) obtained by method C; Figure S4: ^1^H-NMR (a), ^1^H-^1^H COSY (b), ^13^C-NMR (c), HETCOR (d), DEPT (e) and ESI-MS (f) spectra of (5); Figure S5: ^1^H-NMR (a), ^1^H-^1^H COSY (b), ^13^C-NMR (c), DEPT(d) and ESI-MS (e) spectra of (6).

## References

[1] Wright S.L., Kelly F.J. (2017). Plastic and Human Health: A Micro Issue?. Environ. Sci. Technol..

[2] Verma R., Vinoda K.S., Papireddy M., Gowda A.N.S. (2016). Toxic Pollutants from Plastic Waste—A Review. Procedia Environ. Sci..

[3] Nizami A.-S. https://www.bioenergyconsult.com/recycling-waste-to-energy-saudi-arabia/.

[4] Bornscheuer U.T. (2016). Feeding on plastic. Science.

[5] Rahimi A., García J.M. (2017). Chemical recycling of waste plastics for new materials production. Nat. Rev. Chem..

[6] Sharuddin S.D.A., Abnisa F., Daud W.M.A.W., Aroua M.K. (2016). A review on pyrolysis of plastic wastes. Energy Convers. Manag..

[7] Vollmer I., Jenks M.J.F., Roelands M.C.P., White R.J., Van Harmelen T., De Wild P., Van Der Laan G.P., Meirer F., Keurentjes J.T.F., Weckhuysen B.M. (2020). Beyond Mechanical Recycling: Giving New Life to Plastic Waste. Angew. Chem. Int. Ed..

[8] Hou Q., Zhen M., Qian H., Nie Y., Bai X., Xia T., Rehman M.L.U., Li Q., Ju M. (2021). Upcycling and catalytic degradation of plastic wastes. Cell Rep. Phys. Sci..

[9] Wu H.-S. (2021). Strategic Possibility Routes of Recycled PET. Polymers.

[10] Hong M., Chen E.Y.-X. (2017). Chemically recyclable polymers: A circular economy approach to sustainability. Green Chem..

[11] Hoang C.N., Dang Y.H. (2013). Aminolysis of poly(ethylene terephthalate) waste with ethylenediamine and characterization of α,ω-diamine products. Polym. Degrad. Stab..

[12] Ghaemy M., Mossaddegh K. (2005). Depolymerisation of poly(ethylene terephthalate) fibre wastes using ethylene glycol. Polym. Degrad. Stab..

[13] Pingale N.D., Palekar V.S., Shukla S.R. (2010). Glycolysis of postconsumer polyethylene terephthalate waste. J. Appl. Polym. Sci..

[14] Tawfik M.E., Ahmed N.M., Eskander S.B. (2011). Aminolysis of poly(ethylene terephthalate) wastes based on sunlight and utilization of the end product [bis(2-hydroxyethylene) terephthalamide] as an ingredient in the anticorrosive paints for the protection of steel structures. J. Appl. Polym. Sci..

[15] Maurya A., Bhattacharya A., Khare S.K. (2020). Enzymatic Remediation of Polyethylene Terephthalate (PET)–Based Polymers for Effective Management of Plastic Wastes: An Overview. Front. Bioeng. Biotechnol..

[16] Demarteau J., Olazabal I., Jehanno C., Sardon H. (2020). Aminolytic upcycling of poly(ethylene terephthalate) wastes using a thermally-stable organocatalyst. Polym. Chem..

[17] Al-Salem S.M., Lettieri P., Baeyens J. (2009). Recycling and recovery routes of plastic solid waste (PSW): A review. Waste Manag..

[18] Sinha V., Patel M.R., Patel J.V. (2010). Pet Waste Management by Chemical Recycling: A Review. J. Polym. Environ..

[19] Al-Sabagh A., Yehia F., Eshaq G., Rabie A., ElMetwally A. (2016). Greener routes for recycling of polyethylene terephthalate. Egypt. J. Pet..

[20] Park S.H., Kim S.H. (2014). Poly (ethylene terephthalate) recycling for high value added textiles. Fash. Text..

[21] George N., Kurian T. (2014). Recent Developments in the Chemical Recycling of Postconsumer Poly(ethylene terephthalate) Waste. Ind. Eng. Chem. Res..

[22] Sadeghi G.M.M., Shamsi R., Sayaf M. (2011). From Aminolysis Product of PET Waste to Novel Biodegradable Polyurethanes. J. Polym. Environ..

[23] Hoang C.N., Dang Y.H., Pham C.T., Hoang D. (2020). Synthesis of Novel Thermostable Polyamideimides from Bis(2-aminoethyl)terephthalamide and Dianhydrides. ACS Omega.

[24] Chan K., Zinchenko A. (2021). Conversion of waste bottles’ PET to a hydrogel adsorbent via PET aminolysis. J. Environ. Chem. Eng..

[25] Chen Y.-H., Ranganathan P., Lee Y.-H., Rwei S.-P. (2021). New Strategy and Polymer Design to Synthesize Polyamide 66 (PA66) Copolymers with Aromatic Moieties from Recycled PET (rPET). ACS Sustain. Chem. Eng..

[26] Gupta P., Bhandari S. (2019). Chemical Depolymerization of PET Bottles via Ammonolysis and Aminolysis. Recycling of Polyethylene Terephthalate Bottles.

[27] Barnard E., Arias J.J.R., Thielemans W. (2021). Chemolytic depolymerisation of PET: A review. Green Chem..

[28] Gupta K.C., Sutar A.K. (2008). Catalytic activities of Schiff base transition metal complexes. Coord. Chem. Rev..

[29] Andruh M. (2015). The exceptionally rich coordination chemistry generated by Schiff-base ligands derived from o-vanillin. Dalton Trans..

[30] Cozzi P.G. (2004). Metal–Salen Schiff base complexes in catalysis: Practical aspects. Chem. Soc. Rev..

[31] Chen Z., Hay J., Jenkins M. (2012). FTIR spectroscopic analysis of poly(ethylene terephthalate) on crystallization. Eur. Polym. J..

