# Supplementary material for: From Waste to Schiff Base: Upcycling of Aminolysed Poly(ethylene terephthalate) Product
Source: Polymers (Basel). 2022 May 2;14(9):1861. doi: 10.3390/polym14091861 (PMC9100055; doi:10.3390/polym14091861)
Supplement: Supplementary file 1 [file polymers-14-01861-s001.zip › polymers-1646486-supplementary.pdf]

From waste to Schiff base: Upcycling of aminolysed poly(ethylene terephthalate) product

Ahmed A. Al Otaibi[1], Abdulmohsen Khalaf Dhahi Alsukaibi[1,*], Md. Ataur Rahman[2,*] Md. Mushtaque[3] and Ashanul Haque[1,*]

[1] Department of Chemistry, College of Science, University of Hail, Ha'il 81451, Saudi Arabia; a.alotaibi@uoh.edu.sa (AO); a.haque@uoh.edu.sa (AH); a.alsukaibi@uoh.edu.sa (AKDA).

[2] Experimental Research Building, Department of Chemistry, New York University Abu Dhabi, Abu Dhabi P.O. Box 129188, United Arab Emirates; marahmanpub@gmail.com (MAR)

[3] Department of Chemistry, School of Physical and Molecular Sciences, Al-Falah University, Dhauj, Faridabad, Haryana, 121004, India; (mush.chem@gmail.com)

Supporting Information

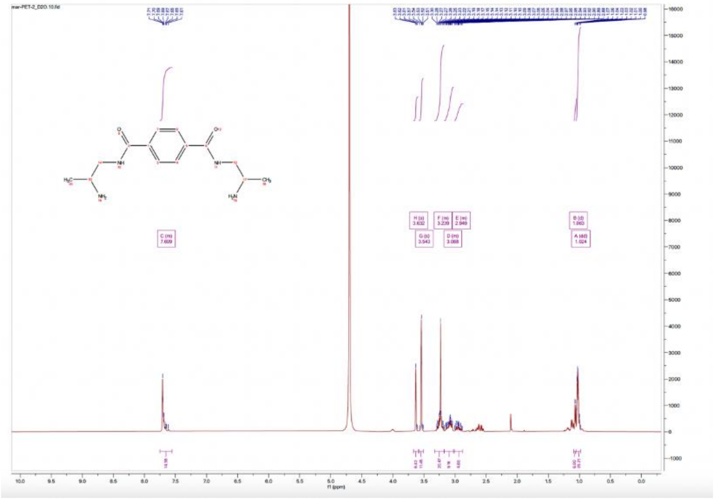

(a)

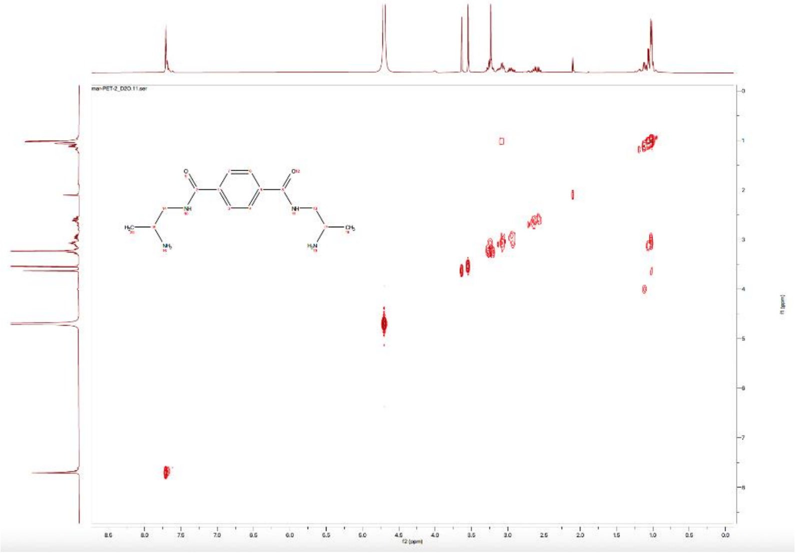

(b)

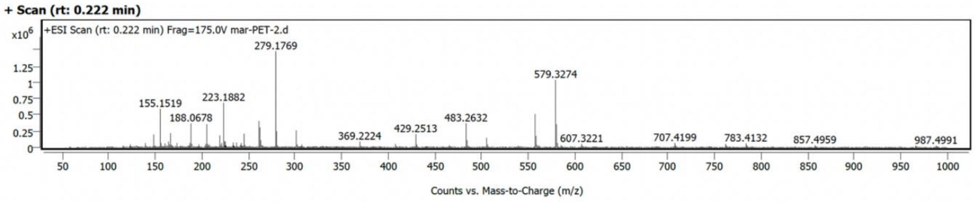

(c)

Figure S1. ^{1}H-NMR (a), ^{1}H-^{1}H COSY (b) and ESI-MS (c) spectra of **(3)** obtained by method A.

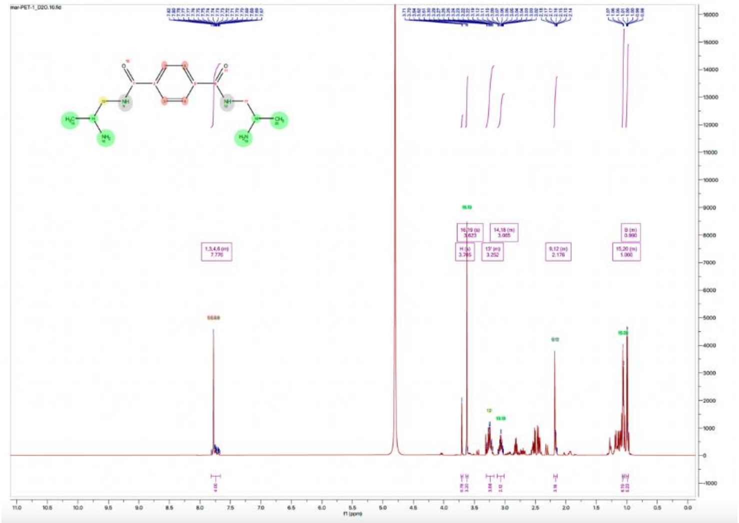

(a)

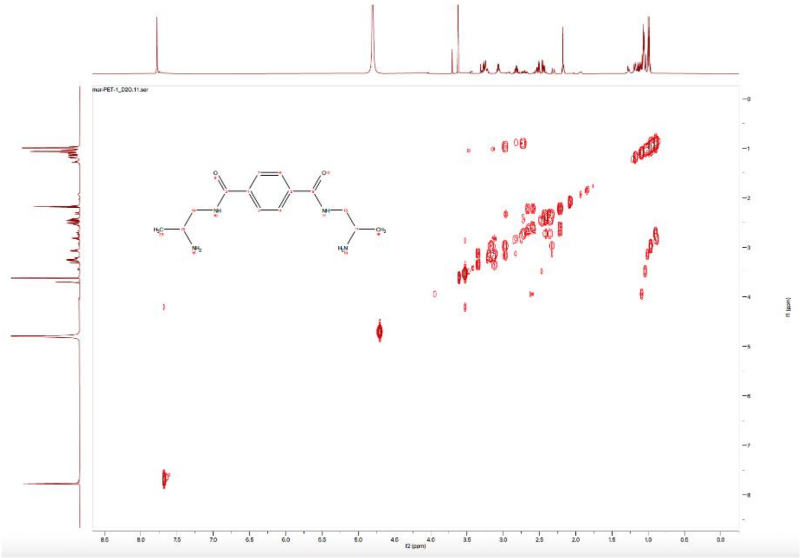

(b)

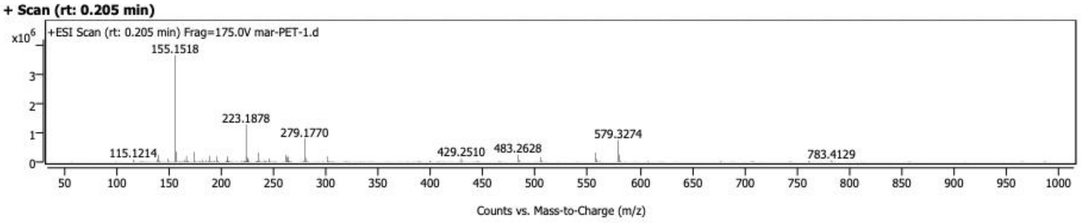

(c)

Figure S2. ^{1}H-NMR (a), ^{1}H-^{1}H COSY (b) and ESI-MS (c) spectra of **(3)** obtained by method B.

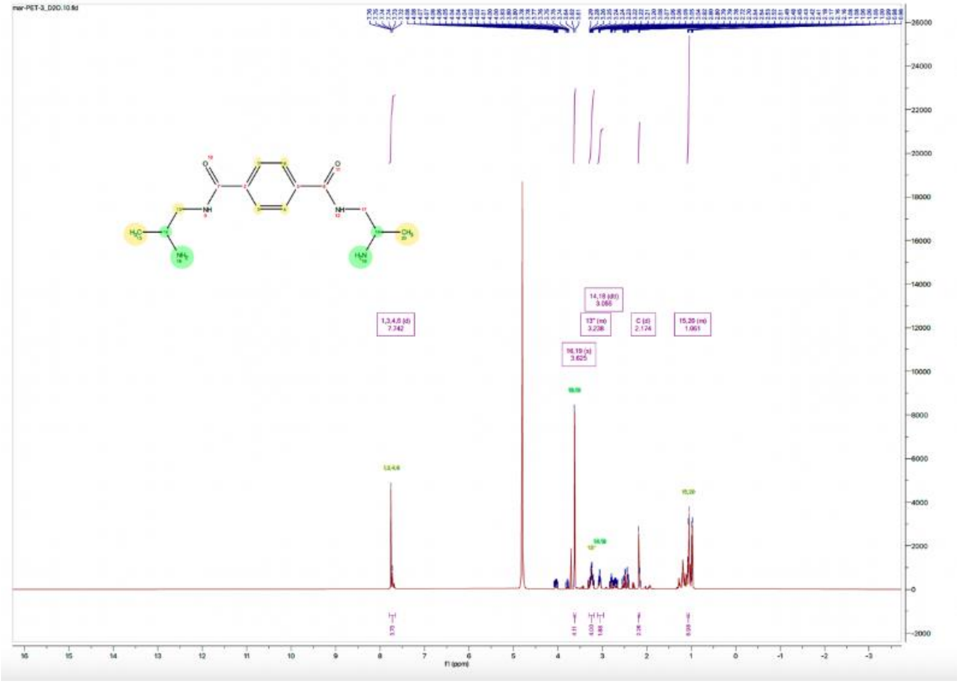
(a)

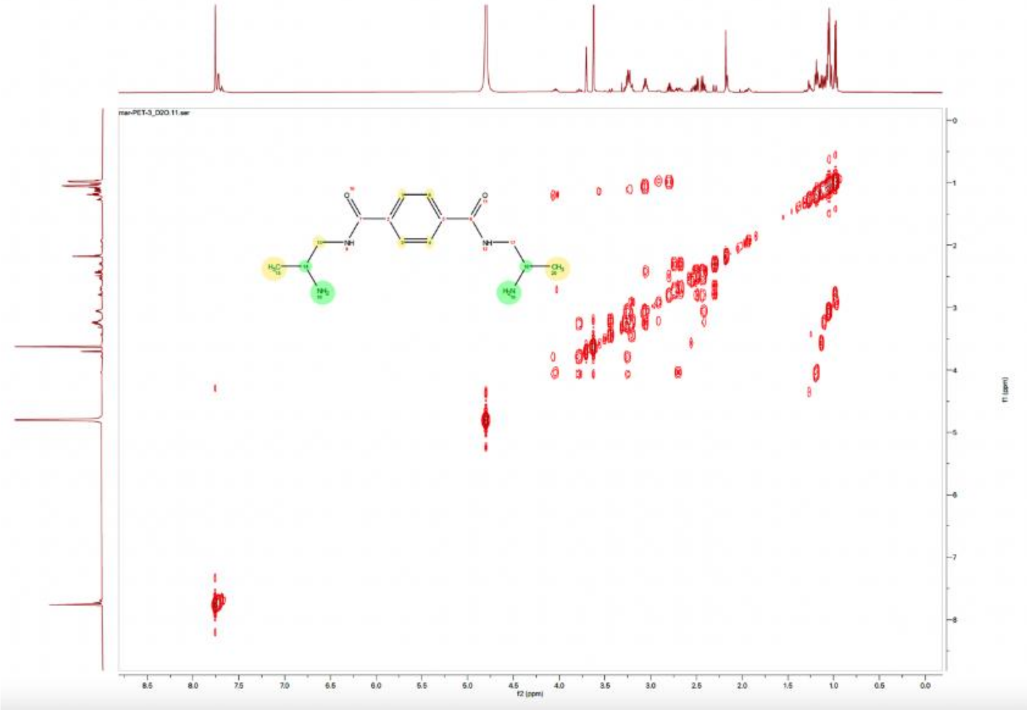
(b)

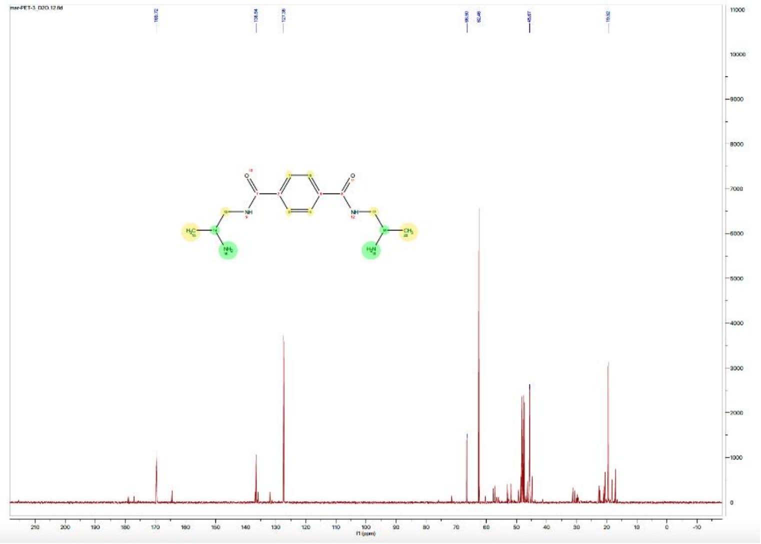

(c)

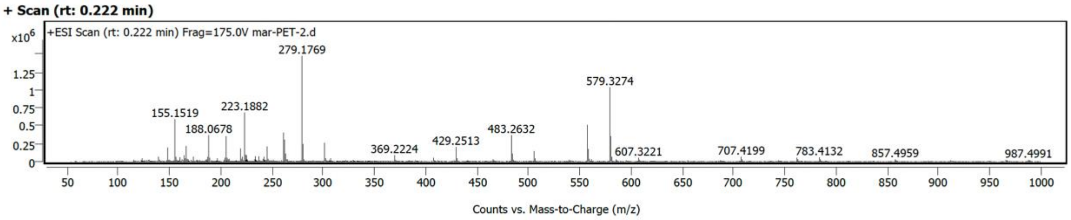

(d)

Figure S3. ^{1}H-NMR (a), ^{1}H-^{1}H COSY (b), (c) ^{13}C and ESI-MS (c) spectra of **(3)** obtained by method C.

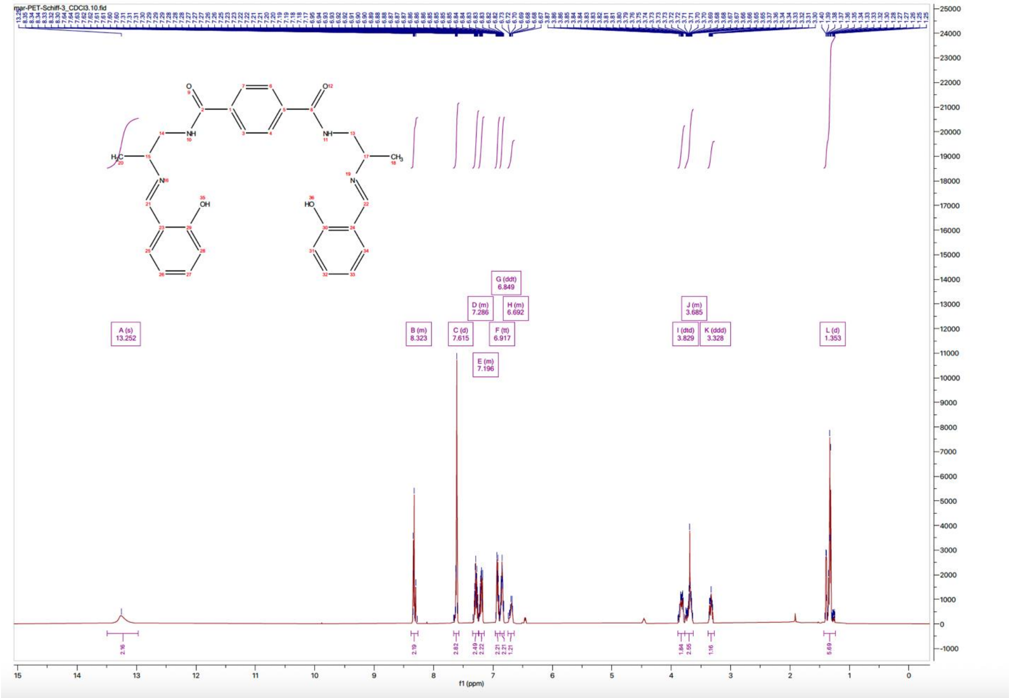
mar-PET-Schiff-3_CDCl3.10.fid
A (s)
13.252
B (m)
8.323
C (d)
7.615
D (m)
7.286
E (m)
7.196
F (tt)
6.917
G (ddt)
6.849
H (m)
6.692
I (dtd)
3.829
J (m)
3.685
K (ddd)
3.328
L (d)
1.353
f1 (ppm)

(a)

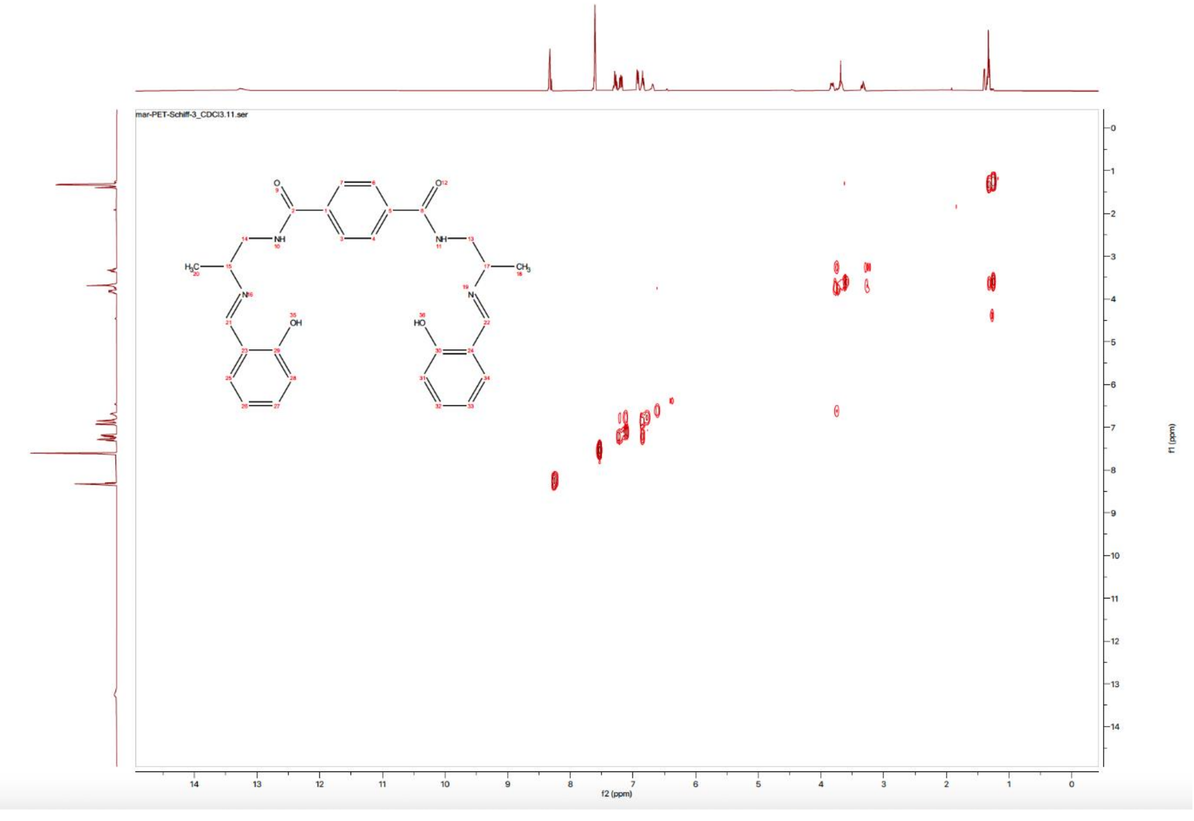
mar-PET-Schiff-3_CDCl3.11.ser
f2 (ppm)
f1 (ppm)

(b)

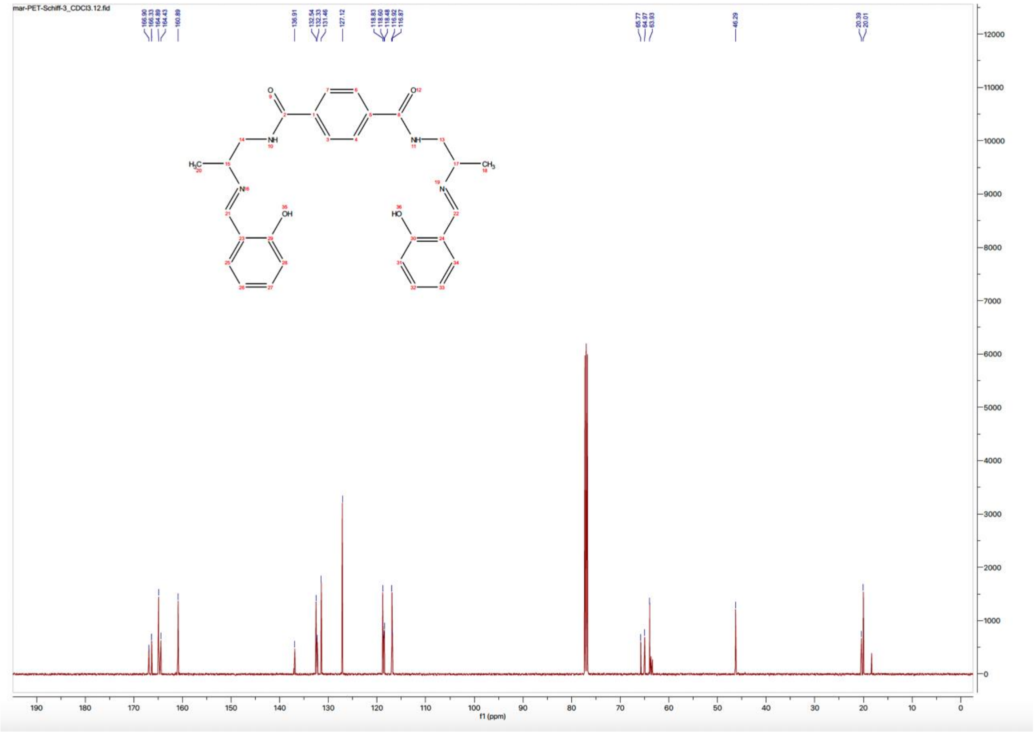
mar-PET-Schiff-3_CDCl3.12.fid
166.90
166.33
164.89
164.43
160.89
136.91
132.54
132.33
131.46
127.12
118.83
118.60
118.48
116.92
116.87
65.77
64.97
63.93
46.29
20.39
20.01
f1 (ppm)

(c)

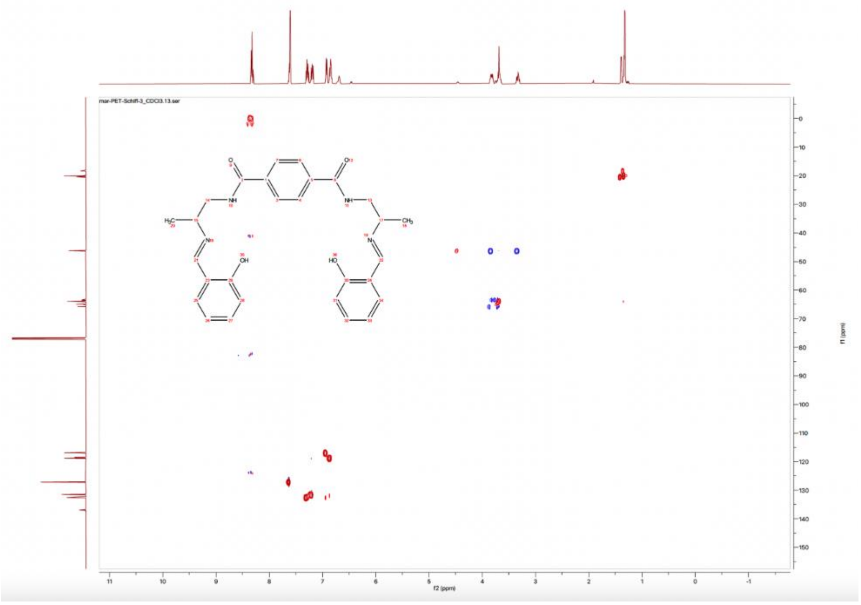
mar-PET-Schiff-3_CDCl3.13.ser
f2 (ppm)
f1 (ppm)

(d)

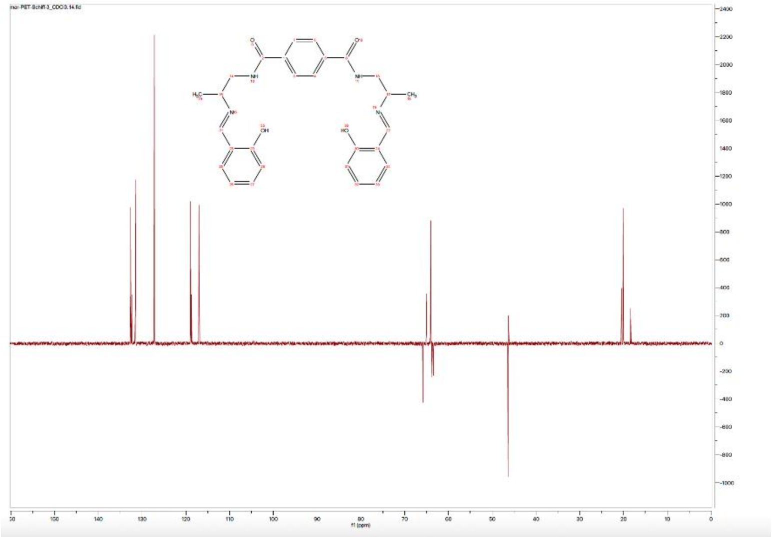

(e)

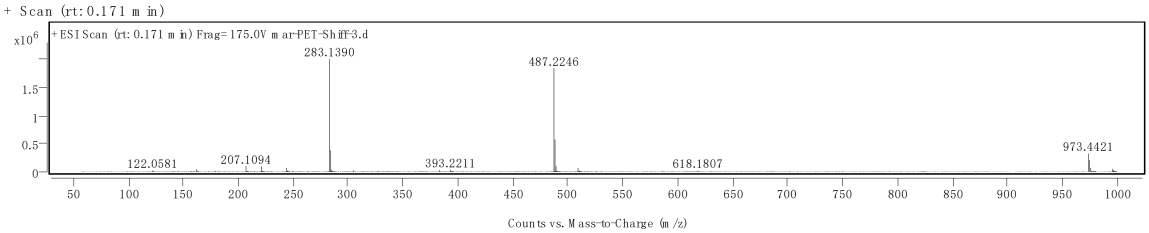

(f)

Figure S4. ^{1}H-NMR (a), ^{1}H-^{1}H COSY (b), ^{13}C-NMR (c), HETCOR (d), DEPT (e) and ESI-MS (f) spectra of **(5)**.

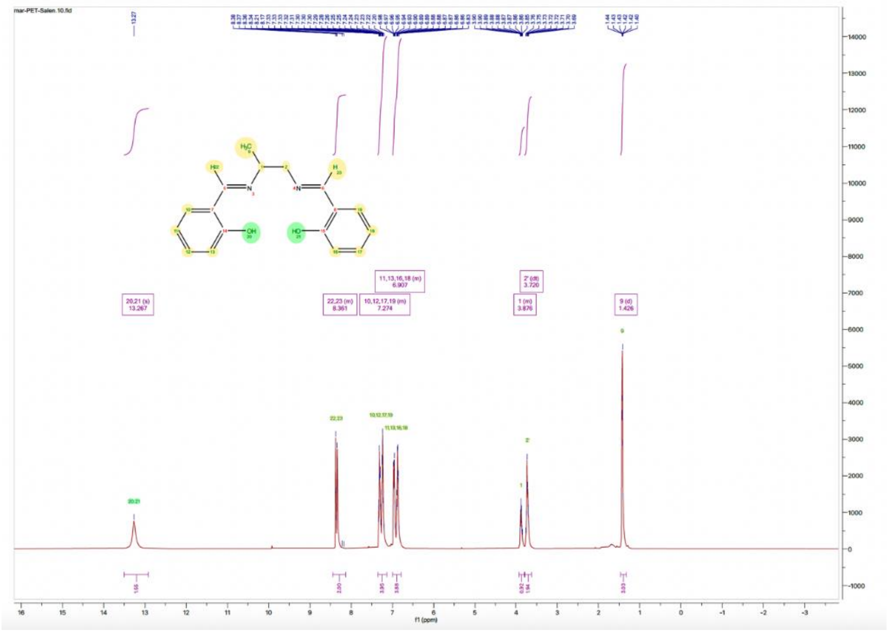
mar-PET-Salen.10.fid
20,21 (s)
13.267
22,23 (m)
8.361
10,12,17,19 (m)
7.274
11,13,16,18 (m)
6.907
2' (dd)
3.720
1 (m)
3.876
9 (d)
1.426
f1 (ppm)

(a)

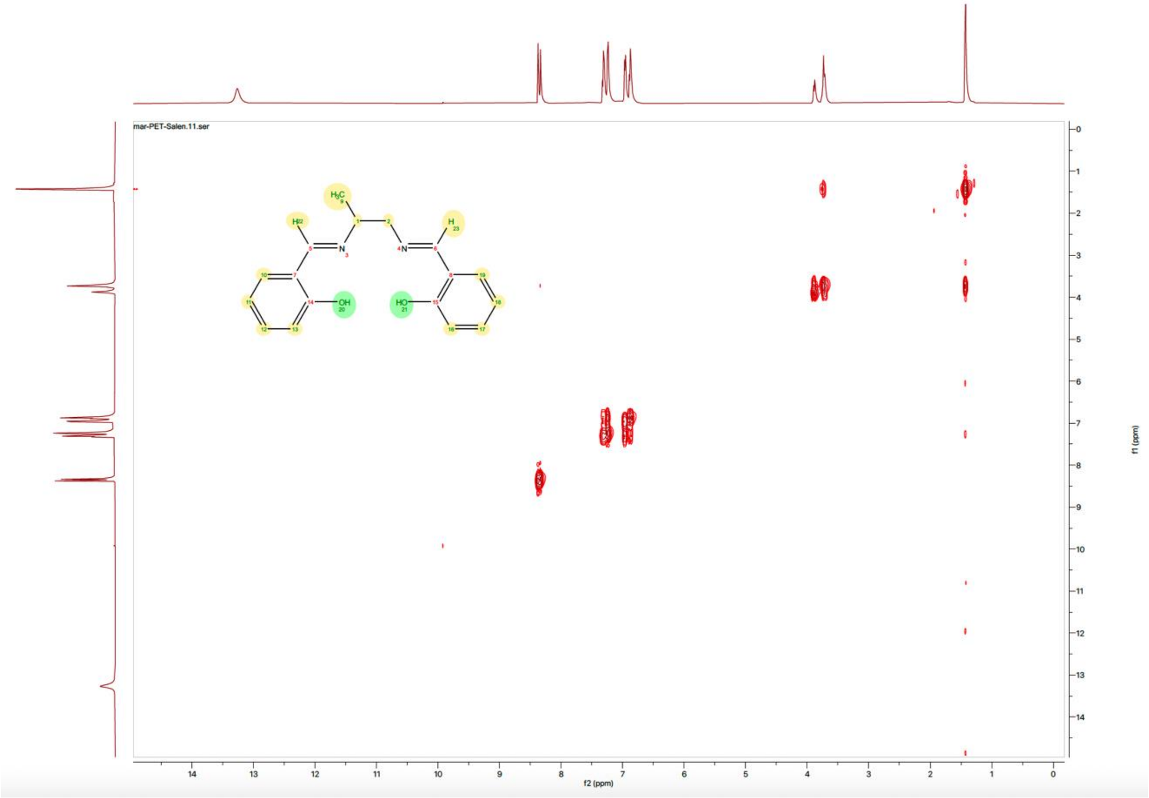
mar-PET-Salen.11.ser
f2 (ppm)
f1 (ppm)

(b)

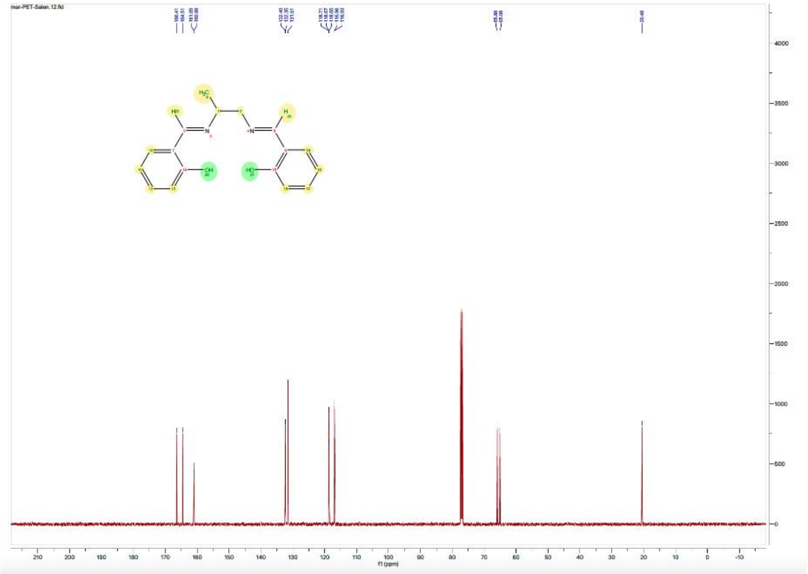

(c)

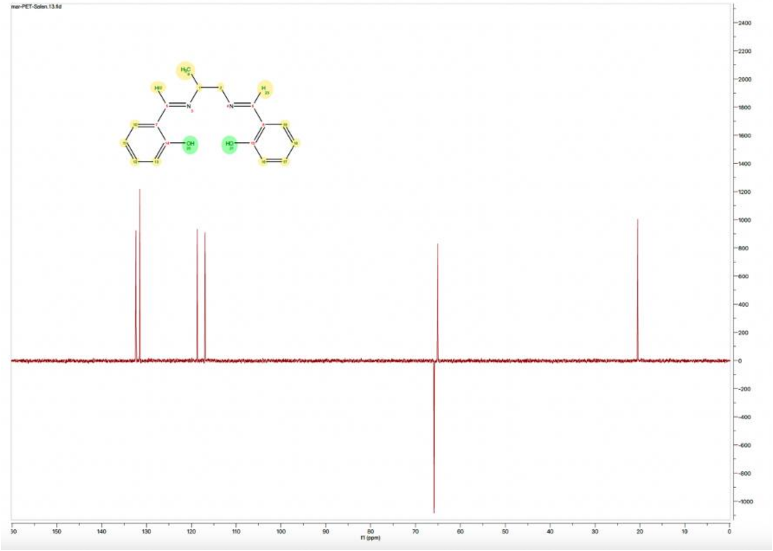

(d)

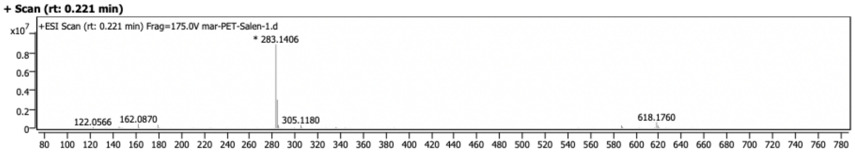

(e)

Figure S5. ^{1}H-NMR (a), ^{1}H-^{1}H COSY (b), ^{13}C-NMR (c), DEPT(d) and ESI-MS (e) spectra of **(6)**.
